# A *Toxoplasma gondii* thioredoxin with cell adhesion and antioxidant function

**DOI:** 10.3389/fcimb.2024.1404120

**Published:** 2024-08-15

**Authors:** Dawei Wang, Yuyi Shi, Ziwen Cheng, Like Luo, Kuo Cheng, Shengqi Gan, Che Liu, Zeliang Chen, Baoling Yang

**Affiliations:** ^1^ College of Animal Husbandry and Veterinary Medicine, Jinzhou Medical University, Jinzhou, Liaoning, China; ^2^ Collaborative Innovation Center for Prevention and Control of Zoonoses, Jinzhou Medical University, Jinzhou, Liaoning, China; ^3^ College of Basic Medicine, Jinzhou Medical University, Jinzhou, Liaoning, China

**Keywords:** Toxoplasma gondii, thioredoxin, glycosaminoglycan, cell adhesion, antioxidant

## Abstract

**Background:**

*Toxoplasma gondii* (*T. gondii*) is a widespread, zoonotic protozoan intracellular parasite with a complex life cycle, which can cause toxoplasmosis, a potentially serious disease. During the invasion process, *T. gondii* proteins first bind to the relevant host cell receptors, such as glycosaminoglycan molecule (GAG-binding motif), which is one of the main receptors for parasites or virus to infect host cells. However, research on TGME49_216510 (*T. gondii* Trx21), a protein from *Toxoplasma gondii*, is limited.

**Methods:**

Bioinformatics analysis of the Trx21 protein was performed firstly. And specific primers were then designed using the conserved domain and GAG-binding motif to amplify, express, and purify a fragment of the Trx21 protein. The purified Trx21-GST protein was used for antioxidant and cell adhesion experiments. Simultaneously, mice were immunized with Trx21-His to generate specific polyclonal antibodies for subcellular localization analysis.

**Results:**

The Trx21 protein, consisting of 774 amino acids, included a transmembrane region, three GAG-binding motifs, and a Thioredoxin-like domain. The recombinant Trx21-His protein had a molecular mass of about 31 kDa, while the Trx21-GST protein had a molecular mass of about 55 kDa, which was analyzed by SDS-PAGE and Western blot. Subcellular localization analysis by IFA revealed that Trx21 is predominantly distributed in the cytoplasm of *T. gondii*. Furthermore, Trx21 exhibited a protective effect on supercoiled DNA against metal-catalyzed oxidation (MCO) and demonstrated adhesion abilities to Vero cells.

**Conclusions:**

These results indicate that Trx21 plays an important role in host cell interaction and oxidative damage.

## Introduction

*Toxoplasma gondii* (*T. gondii*) is a protozoan parasite responsible for causing toxoplasmosis, which infects approximately one-third of the world’s population (Wei et al., 2021). *T. gondii* can invade the nucleated cells of various mammals, birds and vertebrates, including humans. Transmission of *T. gondii* can occur in a variety of ways, such as eating undercooked or raw contaminated meat, drinking contaminated water, handling infected cat litter, or contact with contaminated soil. In addition, the infection can be transmitted from a newly infected mother to her unborn child. Most people with normal immune systems experience latent infection without obvious clinical symptoms. However, people with weakened immune systems, such as HIV/AIDS patients or organ transplant recipients, may experience more serious complications from *T. gondii* reactivation (Attias et al., 2020; Weiss and Kim, 2020; Matta et al., 2021; Tong et al., 2021).

Thioredoxin (Trx) is a group of small redox proteins with a molecular mass of 12 kDa that are known for their widespread distribution, high conservation and importance in various organisms, including mammals (Ghareeb and Metanis, 2020; Masutani, 2022; Zhang et al., 2023). Trx plays a crucial role in antioxidant function by facilitating the reduction of other proteins through cysteine-thiol-disulfide exchange. The dithiol disulfide active site, which contains two adjacent cysteines in a CXXC motif, is found in many organisms, from plants and bacteria to mammals. These cysteines are essential for Trx to carry out protein reduction (Ershov et al., 2022; Hillier et al., 2022; Onodera et al., 2022). The thioredoxin system (Trx system), which consists of Trx, thioredoxin reductase (TrxR), and reduced nicotinamide adenine dinucleotide phosphate (NADPH), is present in eukaryotes and prokaryotes. This system remains reduced through an NADPH-dependent reaction (Bjørklund et al., 2021, 2022; Mohammadi et al., 2019; Jastrząb and Skrzydlewska, 2021; Hasan et al., 2022; Zhang J et al., 2021). By facilitating electron transfer, the Trx system catalyzes redox reactions, which play a crucial role in regulating the activity of biological macromolecules and maintaining oxidative balance in organisms (Suhail et al., 2020; Le Gal et al., 2021; Jeong et al., 2022). Recent studies have shown that *Plasmodium* can be effectively blocked from entering human red blood cells by specific antibodies targeting *Plasmodium* Trx, a heparin-binding protein (Wang et al., 2018).

Invasion of host cells by apicomplexan parasites involves a number of complex processes, including gliding motility, initial attachment, apical attachment, and the formation of moving junctions. The crucial prerequisite for invasion is the adhesion of parasites to host cells (Rastogi et al., 2019; Burrell et al., 2020; Najm et al., 2023; Singer et al., 2023). During this invasion process, specific parasite proteins first bind to host cell receptors such as glycoprotein, lipoprotein, and glycoprotein receptors (Guevara et al., 2020; Yeagle, 2016). Heparin, a glycoprotein receptor widely distributed on cell membranes, is an important glycosaminoglycan molecule (GAG) and one of the primary receptors used by parasites to invade host cells (Tsai et al., 2017; Subramani et al., 2019; Gao et al., 2023). Several publications suggest that heparin molecules can impede parasite invasion into host cells, and the role of heparin-binding proteins in parasites is attracting increasing attention. Several heparin-binding proteins in *T. gondii*, including MIC2, MIC3, ROP2, ROP4, ROP9, SAG1, SAG2 and GRA2, have been identified. These interactions between heparin and parasite surface antigens have been found to contribute to the attachment of *T. gondii* to host cell surfaces and the initiation of the invasion process (Harper et al., 2004; Azzouz et al., 2013; Zhang et al., 2019). In addition, numerous heparin-binding proteins have been identified in *T. gondii* and *Plasmodium falciparum* (*P. falciparum*) by Mass Spectrometry (Zhang et al., 2013; 2014). However, most of these proteins lack functional annotations and further studies are required to determine their importance in the parasite invasion process. One such protein is TGME49_216510, also known as *T. gondii* Trx21, which contains a Thioredoxin-like domain and three GAG-binding motifs. In this study, we investigated the antioxidant and cell adhesion abilities of *T. gondii* Trx21 with the aim of providing a theoretical basis for the development of new therapeutic targets.

## Materials and methods

### Ethics statement

Experiments were performed on female BALB/c mice (6–8 weeks old) purchased from Liaoning Changsheng Biotechnology Company in China. All animals were treated in strict accordance with the ethical guidelines for animal experimentation of the People’s Republic of China. The study was approved by the Animal Ethics Committee of Jinzhou Medical University (Permit Number: SYXK [Liao] 2019–0007).

### Cells and parasites

Vero cells were cultured in 25 cm^2^ culture flasks using DMEM medium (Macgene, China) supplemented with 100 U/mL penicillin, 100 μg/mL streptomycin (Macgene, China) and 10% heat-inactivated fetal bovine serum (FBS) (BI, Israel). The culture was maintained at 37°C in an atmosphere containing 5% CO_2_. *T. gondii* tachyzoites (RH strain) were cultured in Vero cells using DMEM medium supplemented with penicillin, streptomycin, and 2% FBS. The tachyzoites were cultivated at 37°C and 5% CO_2_.

### Bioinformatic analysis of Trx21

We searched the ToxoDB database for genes responsible for encoding thioredoxin in *Toxoplasma gondii*. Our search led us to the identification of TGME49_216510, which encodes a putative protein of 79 kDa. This protein was named Trx21. We then performed an analysis of the signal peptide, transmembrane domain, conserved domain, GAG-binding motif, and antioxidant motif of Trx21.

### Molecular cloning and expression of recombinant Trx21 proteins

Freshly isolated parasites purified by percoll without host cells were collected for Total RNA extraction by TRIzol (Invitrogen, USA) according to the manufacturer’s instructions. DNase I (Takara, China) was used to remove the genomic DNAs completely from RNAs. After that, oligo (dT) primer and reverse transcriptase were employed immediately with reverse transcription carrying out immediately with oligo (dT) primer and reverse transcriptase. PCR with specific primers (Trx21-F: 5’-AAAGGATCCGAAACCCAGGCCGAGGAA-3’; Trx21-R: 5’-TTTGTCGACTTACGCATGCAGTTTCTCCCG-3’) were conducted to obtain the gene encoding fragment of Trx21. The amplified fragment was then cloned into two different vectors, pGEX-4T-1 and pET-28a (Invitrogen, USA). The resulting recombinant plasmids were transformed into *E. coli* BL21 (DE3) for protein expression. To purify the fusion proteins, the GST-tagged and His-tagged Trx21 proteins were purified separately using the Glutathione Sepharose 4B system (GE Healthcare) and the His GraviTrap system (GE Healthcare), respectively, according to the manufacturer’s instructions. The purity and integrity of the purified proteins were assessed using SDS-PAGE and Western blot analysis (Xing et al., 2020).

### Generation of Trx21-specific antibodies

A total of twenty female BALB/c mice were used for the immunization process. Each mouse was immunized with recombinant Trx21-His proteins mixed with Freund adjuvant. The immunization process was repeated a total of four times every two weeks. Antisera were collected 10 days after the last immunization. An enzyme immunoassay (ELISA) was carried out to determine the antibody titers. The recombinant Trx21-GST protein was coated onto microplates for 1 h at 37°C followed by incubation with 5% skim milk (BD, USA) for 1 h at 37°C. Diluted immunized serum samples and untreated serum samples (in dilutions of 1:1000, 1:2000, 1:4000, 1:8000, 1:16,000 and 1:32,000) were added to the microtiter plates and incubated overnight. The microtiter plates were further incubated with HRP-conjugated goat anti-rat IgG (H+L) (1:10,000 dilution, Zsbio, China) for 1 h at 37°C and TMB Chromogen Solution (Beyotime, China) for 15 min. The absorbance was then measured at 450 nm within 1 h in the dark using a microtiter plate reader (Tecan, Switzerland) (Yang et al., 2020).

### Localization analysis of Trx21 by immunofluorescence assay

To prepare samples, purified *T. gondii* tachyzoites were fixed on slides with 4% paraformaldehyde (PFA) for 15 min at room temperature. After fixation, tachyzoites were permeabilized with 0.1% Triton X-100 for 15 min. To block nonspecific binding, the slides were treated with 5% skim milk (BD, USA) at 37°C for 30 min. The slides were then incubated with mouse anti-Trx21 antibody (diluted 1:100) and negative serum (Unimmunized mouse serum, diluted 1:100) for 12 h at 4°C. The slides were then incubated with Alexa Fluor 488-conjugated goat anti-mouse IgG (diluted 1:600, Invitrogen) for 1 h at 37°C. To visualize the parasite nuclei, ProLong Gold Antifade Mountant with DAPI (Invitrogen) was applied to the slides and incubated at 37°C for 30 min in the dark. High-resolution images were captured using a confocal laser scanning microscope (Leica, Germany).

To analyze the localization of Trx21 within the intracellular parasites, the host cells were cultured on slides and then inoculated with freshly isolated parasites purified by percoll. Once the parasitophorous vacuoles became visible (about 20–24 h post-infection), the slides were removed and subjected to the above procedures (Li et al., 2023).

### *In vitro* antioxidant activity assay of Trx21

The Metal-catalyzed oxidation (MCO) DNA cleavage protection assays were conducted with adaptations of previous protocols (Sadat Asadi et al., 2019; Babikir et al., 2021). In these assays, the reaction mixtures consisted of 200 ng pUC19 supercoiled plasmid DNA (Takara, China), 62.5 μM FeCl_3_, 4 mM dithiothreitol (DTT), and different concentrations (ranging from 25 to 800 μg/mL) of purified Trx21-GST recombinant protein. The mixtures were then incubated for 2 h at 37°C in a total volume of 50 μL. GST-Tag (Purification of unmodified pGEX-4T-1 plasmid) protein at a concentration of 800 μg/mL was used as a control. DNA degradation was then assessed by electrophoresis on a 0.8% (w/v) agarose gel containing 0.5 μg/mL ethidium bromide (Godahewa et al., 2019).

### Adhesion assay of recombinant Trx21 with Vero cells

The cell binding activity of Trx21 was examined by Western blot and immunofluorescence assay (IFA). For Western blot analysis, 10 μL of Vero cells were combined with 2 μM soluble recombinant Trx21-GST protein and incubated for 1 h at 37°C according to previously described methods (Li et al., 2022). As a negative control, GST-Tag protein with the appropriate molarity was also incubated with the cells. After incubation, cells were washed three times with PBS and mixed with loading buffer for Western blot analysis. Western blot was performed using an Anti-GST Mouse Monoclonal Antibody as the primary antibody at a dilution of 1:5,000 (TransGen, China). Detection was carried out with the AP-labeled Goat Anti-Mouse IgG (H+L) secondary antibody at dilution of 1:10,000 (Beyotime, China).

In the IFA procedure, all steps were carried out on glass slides. Firstly, Vero cells were harvested with a cell scraper, washed with PBS, and 10 μL of cell precipitate was placed onto the slides. After natural air drying, Vero cells were incubated with either 2 μM soluble recombinant Trx21-GST protein or GST-Tag protein (used as a negative control) for 1 h at 37°C. After incubation, cells were washed three times with PBS and then incubated with 5% skim milk (BD, USA) for 1 h at 37°C. The cells were then incubated with Anti-GST Mouse Monoclonal Antibody diluted at a ratio of 1:5,000 (TransGen, China) for 12 h at 4°C. This was followed by incubation with Alexa Fluor 488-conjugated goat anti-mouse IgG secondary antibody diluted 1:600 (Invitrogen) at 37°C for 30 min. DAPI (Invitrogen) was used to stain the parasite nuclei and incubated for 5–10 min at room temperature in the dark. High-resolution images were captured using a confocal laser scanning microscope (Leica, Germany).

## Results

### Results of bioinformatic analysis

**Figure 1:**
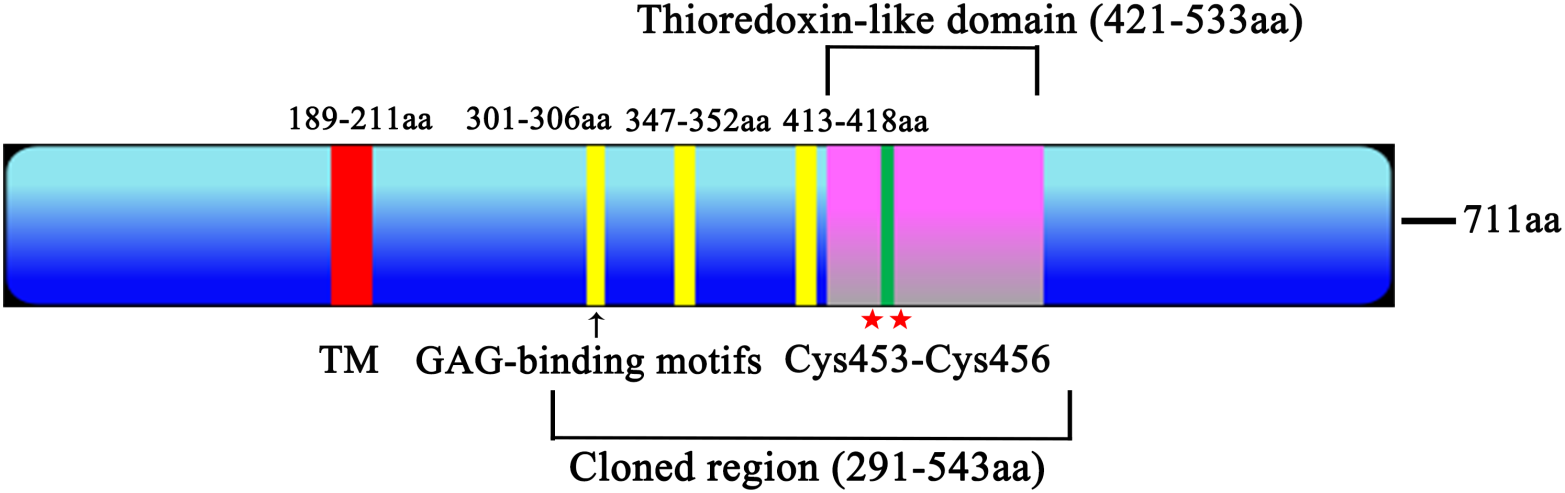
Trx21 functional graph. The Trx21 encoded 711 aa, with a transmembrane region (TM, 189–211 aa), a Thioredoxin-like domain (421–533 aa), two cysteine residues (453 and 456 aa) and three GAG-binding motifs (301–306 aa, 347–352 aa, 413–418 aa). The cloned region (291–543 aa) was a truncated fragment according to the conserved domain and functional motifs.

After screening through the ToxoDB database, a total of 51 *Toxoplasma gondii* thioredoxin proteins were identified and compared with published data, including 21 heparin binding proteins (**Supplementary Table S1**). Comprehensive analysis of 21 heparin binding proteins, including protein size, signal peptides, transmembrane regions, conserved domains, CRISPR Phenotype score and glycosaminoglycan binding motifs, TGME49_216510 was ultimately chosen for further research (low CRISPR Phenotype score, the most GAG-binding motifs and one transmembrane region). The total length of the Trx21 genome is 6648 bp, and the encoded mRNA is 2133 bp, which can be translated into a protein of 711 amino acids. Within the protein structure, there is a transmembrane region spanning amino acid positions 189–211 that lacks signal peptides. In addition, a Thioredoxin-like domain at positions 421–533, and this particular domain contains two cysteine residues, particularly at amino acids 453 and 456. Furthermore, three GAG-binding motifs are present in the protein sequence: one at positions 301–306, another at positions 347–352 and the last at positions 413–418 (**Figure 1**).

### Gene cloning and protein expression of Trx21

**Figure 2:**
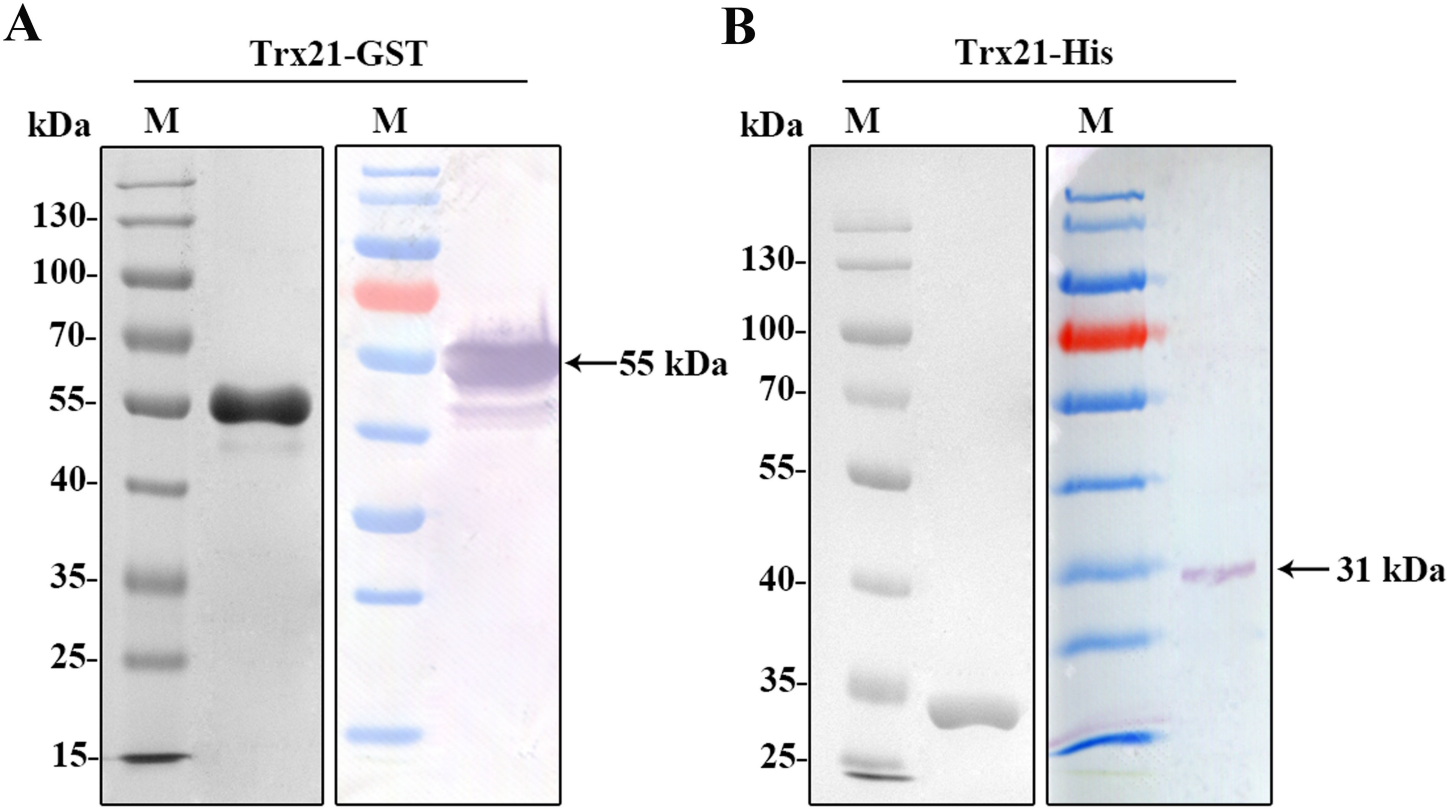
Prokaryotic expression and identification of recombinant protein Trx21. **(A)** Purified Trx21-GST proteins identified by SDS-PAGE and Western blot. **(B)** Purified Trx21-His proteins identified by SDS-PAGE and Western blot.

The coding sequences of Trx21, which was 2133 bp, was shortened into a smaller fragment of 774 bp based on conserved domains and functional motifs in this paper. This truncated fragment was subsequently amplified, and finally PCR product was purified by gel extraction kit (TIANGEN, China) to prepare for digestion (**Supplementary Figure S1A**, **Supplementary Table S2**). T4 ligase was used to ligate the fragments into the pET-28a and pGEX-4T-1 vectors. To validate the ligation, a double enzyme digestion was performed (**Supplementary Figure S1B**). Subsequently, His-tagged and GST-tagged recombinant proteins were expressed. SDS-PAGE analysis and Western blot revealed that the Trx21-GST fusion protein had a molecular mass of about 55 kDa, while the Trx21-His fusion protein had a molecular mass of about 31 kDa (**Figures 2A, B**).

### Localization of Trx21 in T. gondii tachyzoites by IFA

**Figure 3:**
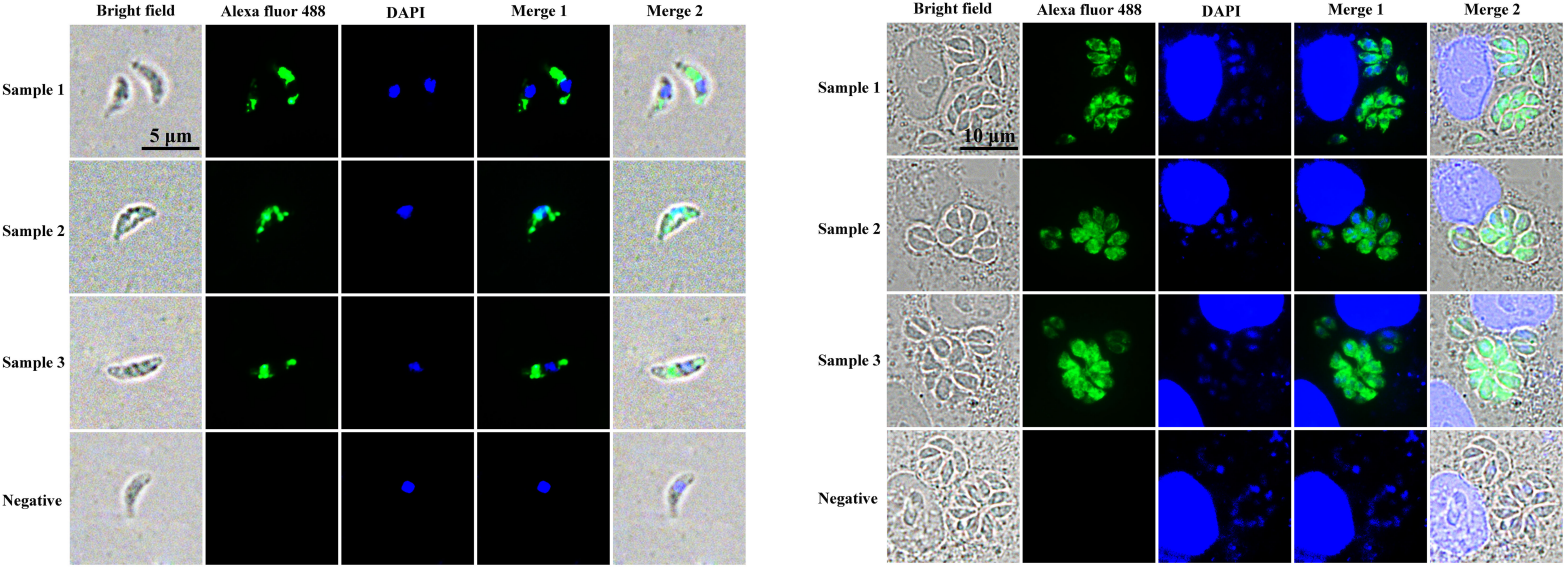
Localization of Trx21 in intracellular and extracellular RH strain tachyzoites. **(A, B)** Localization of Trx21 in extracellular **(A)** and intracellular **(B)** RH strain tachyzoites by IFA. Mouse anti- Trx21 serum and health mouse serum was used as primary antibodies, and Alexa Fluor 488 goat anti-mouse IgG was used as the secondary antibody. Scale bar: 10 μm.

To determine the subcellular localization of Trx21 in *T. gondii*, intracellular and extracellular tachyzoites of RH strain were collected and fixed for IFA. Immunized anti-Trx21 mouse serum was used as the primary antibody (**Supplementary Figure S2**). The results showed that Trx21 (green fluorescence) was widely distributed in the cytoplasm of both intracellular and extracellular tachyzoites (**Figures 3A, B**). However, the fluorescence intensity was observed to be stronger in intracellular tachyzoites than in extracellular tachyzoites.

### Antioxidant activity assay of Trx21

**Figure 4:**
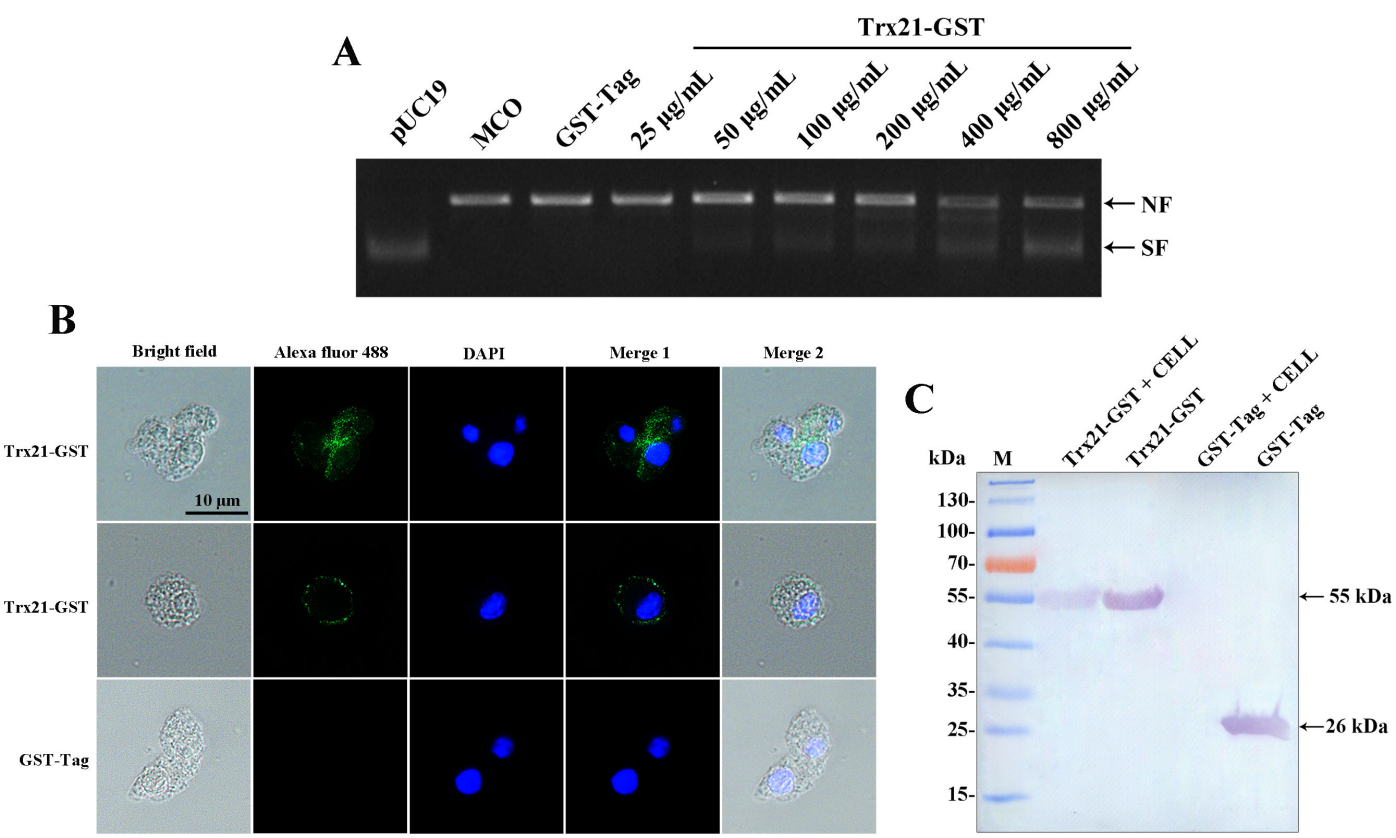
The antioxidant and cell adhesion assay of recombinant Trx21. **(A)** Lane pUC19 represented plasmid alone with no incubation; Lane MCO represented plasmid in the MCO reaction mixture without recombinant protein; The concentrations of Trx-21-GST recombinant proteins ranged from 25 to 800 μg/mL; 800 μg/mL GST-Tag protein was used as control in the MCO reaction mixture. NF: nicked form of the plasmid; SF: supercoiled form of the plasmid. **(B, C)** The adhesion property of *T. gondii* Trx to different cells was detected by Western blot and IFA, respectively. **(B)** For IFA, monoclonal mouse anti-GST was the primary antibody, and Alexa Fluor 488 goat anti-mouse IgG (Invitrogen) was the secondary antibody. Scale bar: 10 μm. **(C)** For Western blot, monoclonal mouse anti-GST was the primary antibody, and AP-conjugated goat anti-mouse IgG was the secondary antibody.

To assess the antioxidant activity of Trx21, a DNA nicking assay was conducted to evaluate its ability to protect DNA from MCO. In the presence of MCO, plasmid DNA in its supercoiled form can be damaged and converted into a linear form. However, the presence of Trx21 may protect against this damage. The extent of DNA damage was determined by observing the shift in gel mobility of pUC19 as it transitioned from its supercoiled to the nicked form. In the MCO system without the recombinant Trx21 protein, the generated reactive oxygen species caused incision of the supercoiled pUC19 DNA (**Figure 4A**). On the other hand, the addition of purified recombinant Trx21-GST protein at a concentration of 25 to 800 μg/mL of the reaction mixture effectively prevented the incision of the supercoiled DNA by the reactive oxygen species. As the concentration of the recombinant Trx21-GST protein increased, the protective effect on pUC19 became more evident (**Figure 4A**). Control experiments with the GST-Tag protein (800 μg/mL) did not show the same DNA protection. Based on these results, it can be concluded that the recombinant protein Trx21 exerts a protective effect on supercoiled DNA against metal-catalyzed oxidation in this assay.

### Adhesion assay of recombinant Trx21 with Vero cells

Recombinant Trx21-GST proteins were exposed to Vero cells and adhesion was assessed by Western blot and IFA. The GST-Tag protein was used as a control. Western blot analysis revealed that recombinant Trx21-GST proteins showed binding to Vero cells, while the GST-Tag protein did not (**Figure 4C**). Additionally, green fluorescence (representing Trx21) was observed on the surface of Vero cells by IFA (**Figure 4B**). Thus, these findings indicate that Trx21 showed affinity for cells.

## Discussion

Thioredoxin is a small, multifunctional protein that is commonly found in a variety range of organisms, including bacteria, plants and animals. Its main function is to regulate the redox state of proteins by facilitating the reduction of disulfide bonds (Muri and Kopf, 2021; Morris et al., 2022). Moreover, thioredoxin plays a crucial role in defending cells against oxidative stress. It can eliminate reactive oxygen species (ROS) and repair proteins damaged by oxidation, thereby maintaining cellular integrity and functionality (Liu et al., 2022). Additionally, Trx is involved in the control of gene expression and signal transduction pathways. By interacting with various cellular proteins, it can modulate their activity and thus influence essential cellular processes such as cell proliferation, apoptosis and the immune response (Tonelli et al., 2018; Tsubaki et al., 2020). In the plant kingdom, Trx is known to regulate crucial functions including photosynthesis, growth, flowering, as well as seed development and germination. In addition, studies suggest its involvement in cell-to-cell communication (Mata-Pérez and Spoel, 2019; Martí et al., 2020). When looking at parasites, there is increasing evidence that Trx is involved in various physiological processes. For example, in *P. falciparum*, Trx functions as a heparin-binding protein, and specific antibodies targeting this protein have shown that they can inhibit merozoite invasion into human erythrocytes. Furthermore, mice immunized with the protein showed significant protection against lethal infection (Wang et al., 2018). Similarly, in *Schistosoma japonicum* (*S. japonicum*), Trx has been identified as a highly abundant protein in adults and is thought to be involved in interactions between the parasite and host cells. Antibodies specifically targeting Trx have shown an anti-*S. japonicum* effect (Angeles et al., 2019; Wanlop et al., 2022). In addition, Trx also plays important roles in *Babesia microti*, *Trypanosoma brucei* and *Wuchereria bancrofti* (Gorai et al., 2022; Kaurov et al., 2022; Piao et al., 2022). However, the characterization of Trx in other parasites is still missing. This study focuses on the analysis of Trx21 in *T. gondii* and demonstrates its potential as a therapeutic target.

*T. gondii* contains a large number of proteins that play different functions throughout its life cycle. Among them, Trx is a major protein family that plays an important role in the invasion and proliferation of *T. gondii*. For example, *T. gondii* Trx4 was a dense granule protein and partially co-localized with GRA1 and GRA5 of *T. gondii*, which knock-out strains resulting in impaired host cell invasion capacity in both RH and Pru strains (Zhang et al., 2024). By contrast, *T. gondii* CTrp26 and CTrx1 were located in the cytoplasm of *T. gondii*, which knock-out strains without influencing the ability of *T. gondii* RH strain to replicate and egress (Zhang ZW et al., 2021). *T. gondii* Trx21 is a transmembrane protein containing the “Thioredoxin-like domain” and three “GAG-binding motifs”. Consensus sequences for “GAG-binding motif” were determined as [-X-B-B-X-B-X-] and [-X-B-B-B-X-X-B-X-], where B is the probability of a basic residue and X is a hydropathic residue (Cardin and Weintraub, 1989). In this study, *T. gondii* Trx21 was truncated into a shorter fragment for protein expression and purification *in vitro* based on the position of the transmembrane region, conserved domain, and functional motifs, which focused on the functions of cell adhesion and antioxidant (**Figure 1**; **Supplementary Figure S1**) (Kogut et al., 2022). The truncated Trx21 was purified by fusing His and GST tags using the *E. coli* prokaryotic expression system, with molecular mass of 31 kDa (His-tagged) and 55 kDa (GST-tagged), respectively. The SDS-PAGE and Western blot results showed that recombinant His-tagged and GST-tagged proteins were detected in specific bands, indicating high specificity (**Figure 2**). For preparation of Trx21 polyclonal antibodies, BALB/c mice were immunized with the recombinant Trx21-His protein and the antibody titers were detected by ELISA test, which met the requirements for further experiments (**Supplementary Figure S2**). Subcellular localization analysis by IFA revealed that *T. gondii* Trx21 was widely distributed in the cytoplasm of *T. gondii*, with the fluorescence intensity being higher in the intracellular compared to the extracellular (**Figure 3**). As thioredoxin, *T. gondii* Thioredoxin reductase and *Plasmodium berghei* Trx are two important proteins for parasites, knocking them out affects parasites proliferation and host interaction (Xue et al., 2017; Gao et al., 2023). Trx21 (TGME49_216510) is an essential gene for *T. gondii* survival as predicted by its low CRISPR-phenotype score of -4.83 (Sidik et al., 2016), and subsequent knockout studies on Trx21 will investigate whether it has similar functions as these two proteins above. After bioinformatics analysis, it was found that there are three GAG-binding motifs on the Trx21 protein. Glycosaminoglycans are a type of heteropolysaccharide mainly present in the tissues and cells of higher animals, including chondroitin sulfate, dermatitis sulfate, keratin sulfate, hyaluronic acid, heparin, and heparin sulfate. Studies have shown that pathogenic proteins containing GAG-binding motifs play an important role in their interactions with the host (Wang et al., 2018; Gao et al., 2023; He et al., 2024; Nagy et al., 2024; Oliveira et al., 2024). To gain further insight into the cell adhesion function of *T. gondii* Trx21, we performed an adhesion assay using recombinant Trx21-GST protein and GST-Tag protein (Negative control) incubated with Vero cells. The results of IFA showed that Trx21 adhered to the surface of Vero cells and exhibits scattered fluorescence distribution, while no fluorescence was observed in the control group (**Figure 4B**). Meanwhile, the Western blot results also demonstrated that Trx21 can adhere to Vero cells (**Figure 4C**). These results indicated a specific interaction between *T. gondii* Trx21 and the glycosaminoglycans of the cell surface by GAG-binding motifs. In the future, the adhesion of *T. gondii* Trx21 to different host cells can be detected, and GAG-binding motifs can also be mutated to observe adhesion function. Thioredoxins (Trxs) function through a redox mechanism in which the reversible oxidation of two cysteine thiol groups to form a disulfide bond enables interaction with a variety range of proteins. Related studies have shown that when the thiol group in two cysteine molecules approaches and undergoes an oxidation reaction, a disulfide bond is formed to connect the two cysteine molecules, thereby resisting external oxidative damage. Besides, this mechanism not only protects organisms from oxidative damage but also confers antioxidant functions (Joardar et al., 2020; Gęgotek and Skrzydlewska, 2023). *T. gondii* Trx21 contains two cysteine residues (453 and 456 aa), which may help *T. gondii* resist external oxidative damage. An *in vitro* assay was performed to evaluate the antioxidant activity of Trx21-GST, the linear form pUC19 plasmids in MCO system are converting to supercoiled form when adding Trx21-GST protein, which revealed that Trx21-GST had a concentration-dependent antioxidant ability (**Figure 4A**). Numerous studies have highlighted the importance of the “-CXXC-” motif (Two adjacent cysteine residues) in the antioxidant function of Trx, while reports suggest that “-CXXS-” motif (Adjacent cysteine and serine) and “-CXXT-” motif (Adjacent cysteine and threonine) also exhibit antioxidant activity (Fomenko and Gladyshev, 2002; Božok et al., 2018, 2003; Hillier et al., 2022). In this study, only “-CXXC-” motif was analyzed, and in the future, this motif can be eliminated or mutated into “-CXXS-” and “-CXXT-” to further analyze its antioxidant function.

## Conclusions

In conclusion, *T. gondii* Trx21, a protein with GAG-binding motifs and thioredoxin domain, plays an important role in host interaction and antioxidant. Subsequently, Trx21 point mutation protein or gene knockout/overexpression strains can be constructed to further investigate protein function, contributing to the advancement of novel *T. gondii* therapeutic targets researches.

## Data Availability

The original contributions presented in the study are included in the article/**Supplementary Material**. Further inquiries can be directed to the corresponding author.

## Supplementary material

Supplementary Figure S1 Trx21-truncated fragment amplification and double enzyme digestion validation. **(A)** PCR product of truncated fragments of Trx21 gene. **(B)** Double enzyme digestion of Trx21-His and Trx21-GST recombinant plasmids.

Supplementary Figure S2 Anti-Trx21 serum antibody titer detection.

Supplementary Table S1 Information of 51 thioredoxin and 21 heparin binding proteins.

Supplementary Table S2 Trx21 full coding sequences and truncated sequences.
