# Supplementary figures and images for: A Toxoplasma gondii thioredoxin with cell adhesion and antioxidant function
Source: Front Cell Infect Microbiol. 2024 Aug 15;14:1404120. doi: 10.3389/fcimb.2024.1404120 (PMC11358088; doi:10.3389/fcimb.2024.1404120)

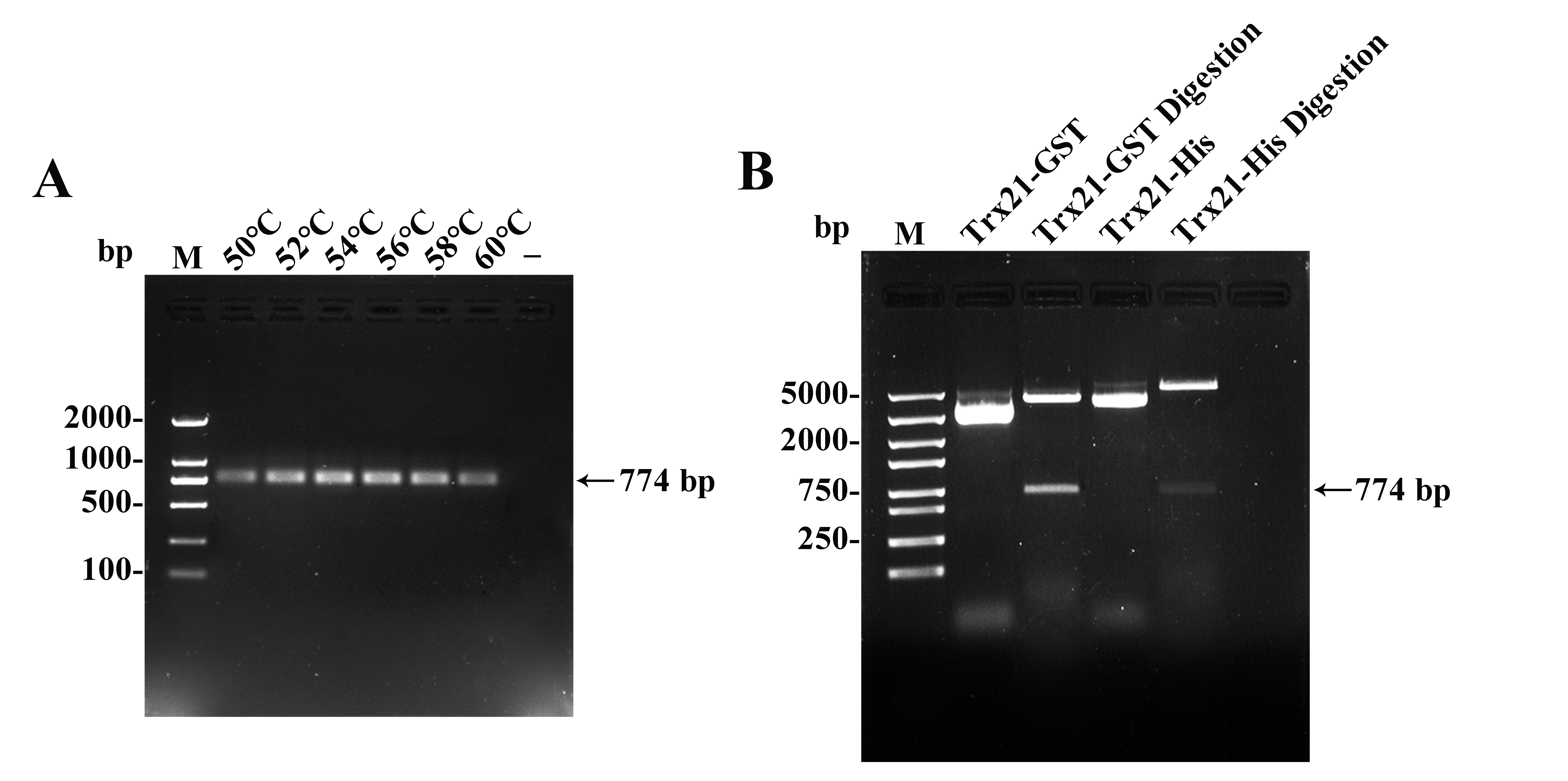

Supplement: Supplementary Figure S1 — Trx21-truncated fragment amplification and double enzyme digestion validation. (A) PCR product of truncated fragments of Trx21 gene. (B) Double enzyme digestion of Trx21-His and Trx21-GST recombinant plasmids. [file Image1.tif]

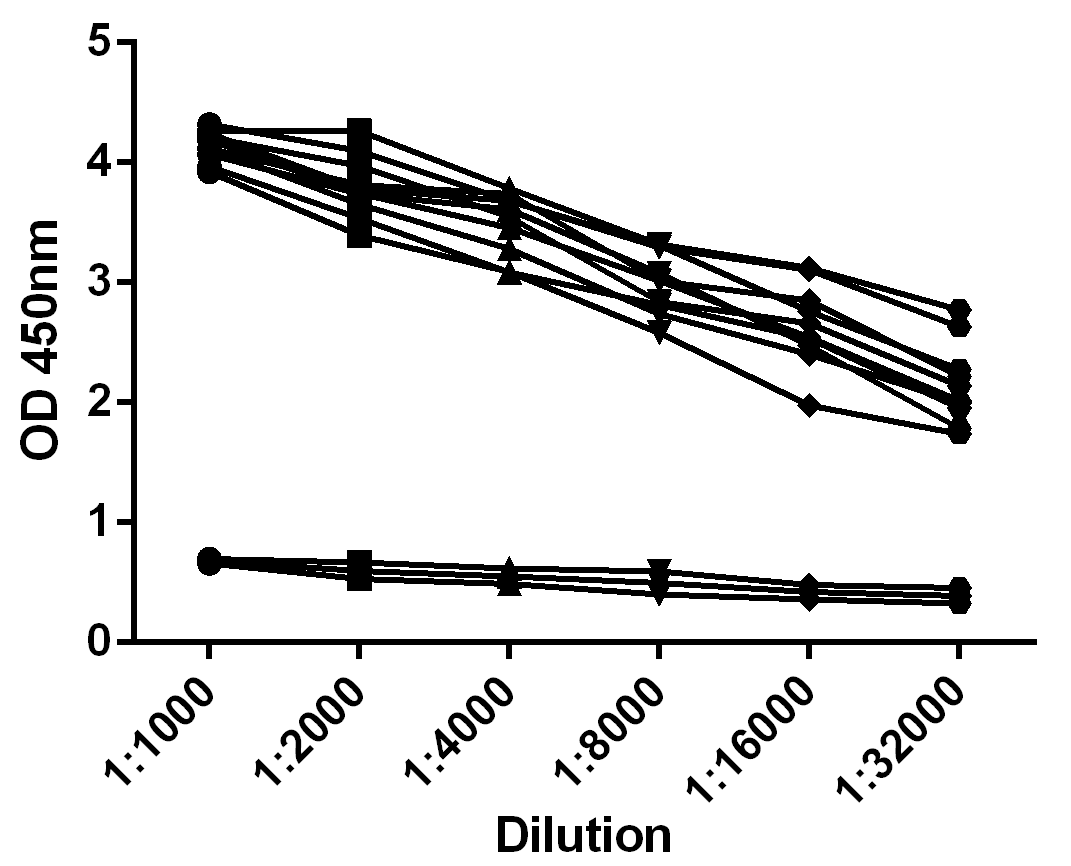

Supplement: Supplementary Figure S2 — Anti-Trx21 serum antibody titer detection. [file Image2.tif]
