# Supplementary material for: A Toxoplasma gondii thioredoxin with cell adhesion and antioxidant function
Source: Front Cell Infect Microbiol. 2024 Aug 15;14:1404120. doi: 10.3389/fcimb.2024.1404120 (PMC11358088; doi:10.3389/fcimb.2024.1404120)
Supplement: Supplementary Table S2 — Trx21 full coding sequences and truncated sequences. [file Table2.docx]

**TGME49_216510 full coding sequences (2136 bp)**

ATGTCGCGCCGCCTTCCGCCTCGCAGTTTCTTCGCCTCTCAGGCGGTCGCTGTCCGCGCCGCTTCTCTCTCGCAGGCGCCTTGCAGATTCCGCGGCTTTGCTGCTTTCTGCAGCGCCTTATCATCGTCTTGTGCAAATTCTGCGCTGCGCTCGCATGCGGTTTCCCTGTGGACCTCGCGGCTCTCGAGTGACAGACGGGTCTCGAATCCGGTTTCCACTGCCGATCGAAACTCCGCAGAACGCGCCCTCAAACGCGATGTGCGTCTCGGCGCTCTGCTCGCGCCTCTCCGCCGAGAAGTTGTGCATGCAGTCGGCTCTGCCGCGAGTCCCAGCAGCCGCGAGAAGAAATTCTTCAGCTCCAGAGCCGGCGAAGGTCGAGACGCCGCGCTCGACAGCGCGTCTCACCCCAACGACGTAGAAATGAAGGAGACCGAGCAGGCGAGCGCGCCGGAGAATGAAGGGAAGAAGAAGGAGAAAGAGACGGAGGGGAGTCGTGGAAAGTCTGAGTCTGTGGGGGCGGGGGAAGCGGTCCACCGAAGCGGTCTGCGGCGCGTGTTTTCGCGGCTGTTCTACTCGTGCGTTCTCCTCGTTGGAGGCGGCGCTGCGTTTCTCTTCTTCGCCTTTCCGTCGGTGCCTCTGGACCTGAAAAACGTGCCGGTTGGGTCCGCGCTGCAGGCCGCGCTGCAGGCCGCGCTGGGTGGGAAGGAGACTCCTGAGGAGACAGGAGACAGGAAGTCGGAGACAGGGGGGAAGGAGACGCGTTCGTCCTTCGCCTTCCTTGTGCGGCGAGATCCCCTCGAAGGATTCTCTCTTGTGACAGACGCAGAATTCGACAAGACGGATGAGGCCCTCGTTCTCTTCCTCGACGGTGAAACCCAGGCCGAGGAAGAGAGCGAAGCCATCCGCAAACTACGCGCTCTTGTCGAACGCATGCAACAGGAAGGAAAACTAAAAAACATCAGACTCTTCTACGCCTGGAGGACGGCAGGGAATTCTCCAGCTCAGGGCGAAGACACGGCAGTGATGCTCTACAAGGGACAGCGTCGGTCGCGGTACGCTCTCGCCGAGCTTTTGCGCGGCGAGGCGGAAGAAGCAGGCGAAGTAGACCCCAAAGACAAGAACGTAGAGCCAGCAGCGCTTGGAGAAGAAAGGACGAGTTCAAGGGAAGCCGCGAGTGGAGAGAAGCTTCTCGAGACATTCTTTACGCCGTTGAGCGAGAAGGAGAACGCGTCGCGCACGAAGCGCGAGAAGGGCAAACATCTGCCCATCCGAGTCGTCGGTTCCGCCTTCAAACGCGATGTCTTGGACGAGGCGAAGGCGGGCAATACGATTCTTCTTCAACTGTTTGAAGACTCTTGTTTCCTCTGCTTCCTCATGCGACCTTTCCTGAACTCGGTCAGCGCACTGCTTGCCGAGTACAACATCCCGGTGACGATGAAGAGGTTGAACATCGAAAAGAACGACTTTCCAGAGGGATGCGTCGTCACCAGGGCGACCCCAACGTTCGTGTTGCACCGAGGCGCACACGAGGAGGGAGAGAGATGGTCAGAATTCCGACCTCGAGATTTCATTGAAAAACTGGAGAAGGAGTTCGACCTGCCTGTTGAGCTGCGGGAGAAACTGCATGCGCTCCTTGACCTCCTGCATGAGCGCTTCAAGCGCTTCGGTCTTCTCTCTGTTTGGTTGCTGGAAGTGCGTAAGATGGAGGAAGCGTTCCTTCAGGAGCAGCAGCGGAAGCAAGCGCGAGAGAGCCTCGAGCTCCACGGCCTCGGCGCGCAGACCGCTTCTTCTTCGCCTTCTTCTTCTTCTGGAAACCATCAGACGGAAGCTCGCGAAAAAACGACAGACGAAAAGTCGAGAGAACAGGACGAGAAGCAACGGGAAGACGGCGACTTCGACGCCATTGTATCGATGCTCATGAGTCAAGACATGAAGCGCTTCGATGACCTCAGTGAAAATCTCGAGCACCTGGAGCGCGAAGCTGCGAACGCAGAGGCCGACGCGATGGCTCTCGGTGTGATGATGGGAGAAGAATTGCTGCGCGACGACGTTGAACATCTCGCGGAAGCTCTGTTGGCAGCTGAGAGTCTCGTCGGACAAAGCTCATCGGTGGAAGGCGGCGCAGGGCGACACTGA

**TGME49_216510 truncated sequences (774 bp)**

AAggatccGAAACCCAGGCCGAGGAAGAGAGCGAAGCCATCCGCAAACTACGCGCTCTTGTCGAACGCATGCAACAGGAAGGAAAACTAAAAAACATCAGACTCTTCTACGCCTGGAGGACGGCAGGGAATTCTCCAGCTCAGGGCGAAGACACGGCAGTGATGCTCTACAAGGGACAGCGTCGGTCGCGGTACGCTCTCGCCGAGCTTTTGCGCGGCGAGGCGGAAGAAGCAGGCGAAGTAGACCCCAAAGACAAGAACGTAGAGCCAGCAGCGCTTGGAGAAGAAAGGACGAGTTCAAGGGAAGCCGCGAGTGGAGAGAAGCTTCTCGAGACATTCTTTACGCCGTTGAGCGAGAAGGAGAACGCGTCGCGCACGAAGCGCGAGAAGGGCAAACATCTGCCCATCCGAGTCGTCGGTTCCGCCTTCAAACGCGATGTCTTGGACGAGGCGAAGGCGGGCAATACGATTCTTCTTCAACTGTTTGAAGACTCTTGTTTCCTCTGCTTCCTCATGCGACCTTTCCTGAACTCGGTCAGCGCACTGCTTGCCGAGTACAACATCCCGGTGACGATGAAGAGGTTGAACATCGAAAAGAACGACTTTCCAGAGGGATGCGTCGTCACCAGGGCGACCCCAACGTTCGTGTTGCACCGAGGCGCACACGAGGAGGGAGAGAGATGGTCAGAATTCCGACCTCGAGATTTCATTGAAAAACTGGAGAAGGAGTTCGACCTGCCTGTTGAGCTGCGGGAGAAACTGCATGCGTAAgtcgacAA
